# A Case Report: The Challenging Diagnosis of Spontaneous Cervical Epidural Hematoma

**DOI:** 10.5811/cpcem.2020.5.47107

**Published:** 2020-07-13

**Authors:** Francis L. Counselman, Julie M. Tondt, Harry Lustig

**Affiliations:** * Eastern Virginia Medical School, Department of Emergency Medicine, Norfolk, Virginia; † Emergency Physicians of Tidewater, Norfolk, Virginia

**Keywords:** Spontaneous cervical epidural hematoma, neck pain, epidural hematoma

## Abstract

**Introduction:**

We present the case of a patient with a spontaneous cervical epidural hematoma that presented with neck pain and mild, left arm parasthesia.

**Case Report:**

A 59-year old man presented with sudden onset of severe neck pain, without history of injury or trauma. The patient also complained of associated left arm parasthesias that progressed to left arm and leg weakness while in the emergency department. Multiple diagnoses were considered and worked up; eventually the correct diagnosis was made with magnetic resonance imaging of the cervical spine.

**Conclusion:**

Spontaneous cervical epidural hematoma typically presents with neck pain, and variable neurologic complaints. This case illustrates the challenge in making this uncommon but serious diagnosis.

## INTRODUCTION

Cervical spinal epidural hematomas can be either spontaneous or secondary to trauma, with the latter much more common. Spontaneous cervical epidural hematomas (SCEH) have been associated with bleeding disorders, vascular abnormalities, or the use of antiplatelet and anticoagulant medications. Typically, these patients present initially with neck pain, which can then progress to parasthesia and weakness as the hematoma expands and compresses the spinal cord. The presentation can easily be confused with a stroke, transient ischemic attack (TIA) or an arterial dissection. Magnetic resonance imaging (MRI) is the study of choice to identify this disease process. Our case is interesting because the only risk factor was daily aspirin 81 milligrams (mg), and the initial presentation was confusing and included a wide differential diagnosis. Only after the development of left-sided weakness was a cervical MRI ordered and the correct diagnosis made.

## CASE PRESENTATION

A 59-year-old man presented to our freestanding emergency department (ED) with abrupt onset of severe, upper back and neck pain. The patient stated he experienced sudden, severe, upper back and neck pain in the shower as he raised his arms over his head to wash his hair. He stated the pain increased with arm movement. He also complained of mild paresthesia in the left arm. He denied weakness, and there was no history of trauma, injury, or overuse. He denied fevers, chills, chest pain, shortness of breath, or headache. Past medical history was significant for hypertension, type 2 diabetes mellitus, peripheral vascular disease, and coronary artery disease. Medications included metformin 1000 mg twice each day, rosuvastatin 20 mg daily, telmisartan-hydrochlorothyazide 80–25mg daily, fenofibrate 145 mg each day, and aspirin 81mg daily. The patient stated he had quit smoking five years prior and consumed alcohol only on occasion.

The patient appeared uncomfortable secondary to pain. Physical exam revealed a pulse of 76 beats per minute, respiratory rate of 15 breaths per minute, blood pressure 156/82 millimeters of mercury, 97% oxygen saturation on room air, and he was afebrile.

The head, eyes, ears, nose, and throat exam was normal. The heart exam was normal, and auscultation of the lungs revealed clear, bilateral breath sounds. The abdomen was soft, nontender, and without guarding or rebound. The patient exhibited tenderness in the lower cervical and upper thoracic region posteriorly, both in the midline and paraspinal region bilaterally. He described increased pain when he lowered his left arm from the abducted position. On initial neurologic examination, the patient exhibited normal strength, without sensory deficits, in all four extremities. He did complain of left arm paresthesia.

An intravenous (IV) line was established, and the patient was administered hydromorphone 0.5 mg and ondansetron 4mg IV for pain. A stat electrocardiogram revealed normal sinus rhythm, normal axis, and no evidence of ischemia or injury. Laboratory studies were sent for a complete blood count (CBC), basic metabolic profile (BMP), urinalysis, and a troponin T.

The emergency physician (EP) was concerned the patient was experiencing a dissecting thoracic aortic aneurysm and ordered a computed tomography angiography (CTA) scan of the chest, abdomen, and pelvis. The CBC was normal, and the BMP was remarkable only for a serum glucose of 355 mg per deciliter (mg/dL) (Reference: 70–99 mg/dL), with a normal serum bicarbonate. The urinalysis and troponin T were normal.

The patient returned from radiology after his chest CTA, complaining of continued pain. He received an additional hydromorphone 0.5 mg IV. While the CT was waiting to be read, change of shift occurred, and the case was turned over to the oncoming EP.

The chest CTA was interpreted as no active aortic pathology, severe coronary arteriosclerosis, and mild to moderate hepatic steatosis. Given the lack of an identifiable cause of the patient’s severe pain, the oncoming EP performed his own history and physical exam. The patient now complained of new left arm and leg weakness, in addition to the left arm paresthesia. On exam, the patient had 2/5 motor strength in the left leg, and 4/5 motor strength in the left arm. He had strong pulses in all four extremities. The oncoming EP expanded the differential diagnosis to include carotid artery dissection, cerebrovascular accident, and TIA. After discussion with radiology, a low-dose CTA of the head and neck was ordered.

The CTA of the head and neck was interpreted as “no acute arterial disease identified, and no dissection of carotid artery (or any other artery). There is no acute thromboembolism or flow limiting stenosis identified.” Given the negative CTA and abnormal neurologic exam, an MRI of the cervical spine, with and without contrast, was ordered. The patient required two more additional doses of hydromorphone 0.5 mg IV for pain, and was administered IV normal saline at 125 cubic centimeter per hour.

Radiology called the ED immediately after reviewing the MRI. They described a “moderate sized T1 isointense T2 hyperintense left posterolateral epidural collection, extending from the second and third cervical (C2/C3) through mid C7 level, without internal or thick peripheral enhancement.” See Image. Given the history, this was thought to reflect a hyperacute (less than 12 hours old) cervical epidural hematoma.

**CPC-EM Capsule**

What do we already know about this clinical entity?

Spontaneous cervical epidural hematoma is a rare disease process, much less common than traumatic spinal epidural hematoma. While associated with antithrombotic medications, bleeding disorders and vascular abnormalities, in a significant number of cases, the cause remains unknown.

What makes this presentation of disease reportable?

The only risk factor for our patient was the daily use of aspirin 81mg. In addition, while initially complaining only of associated parasthesias, his neurologic exam rapidly deteriorated.

What is the major learning point?

For patients complaining of neck pain and any associated neurologic symptoms, spontaneous cervical epidural hematoma should be included in the differential diagnosis. Magnetic resonance imaging is the study of choice.

How might this improve emergency medicine practice?

Timely diagnosis and appropriate treatment can help prevent devastating neurological injuries.

The EP immediately consulted neurosurgery at a nearby hospital for transfer and admission. The patient was accepted by neurosurgery and transported via air ambulance to the receiving hospital. The patient’s neurologic exam remained unchanged, with the noted left-sided weakness. Coagulation studies were ordered prior to taking the patient to the operating room (OR). The protime, international normalized ratio and partial thromboplastin time were all normal. However, the platelet function assay was elevated, thought to be secondary to the patient’s daily aspirin use. The patient was transfused one unit of platelets IV and taken to the OR where he underwent a left C3–C6 hemilaminectomy for evacuation and drainage of the epidural hematoma. During the surgery, they found a sizable, left epidural hematoma causing spinal cord and nerve root compression from C3 through C6, on the left side only.

The postoperative course was uneventful. The paresthesia resolved, and the patient gradually regained his strength. He was discharged on postop day six, with a hard cervical collar. He was seen in follow-up eight days later with a completely normal neurologic exam.

## DISCUSSION

Cervical spinal epidural hematomas are an uncommon neurologic emergency that can broadly be categorized as either spontaneous or traumatic. Spontaneous spinal epidural hematomas were first described in the literature in 1869 by Jackson, and have since been reported to have an incidence of 1 per 100,000 people per year and represents 0.3–0.9% of all spinal cord lesions.^1^,^2^ In comparison, traumatic spinal epidural hematomas have been reported to occur in 0.5–1.7% of all spinal injuries.^3^ Spinal epidural hematomas occur within the epidural space, similarly to those occurring within the cranium, and cause compression on the vasculature and cord within the spinal canal.

SCEH have been attributed to various causes, including bleeding disorders, antithrombotic medications, or vascular abnormalities.^4^,^5^ Aspirin, warfarin, rivaroxaban, and dabigatran are a few of the antithrombotic (antiplatelet, anticoagulant) medications reported to have been used by patients ultimately diagnosed with SCEH. Emamhadi et al described a case of a 77-year-old woman on aspirin and antihypertensive medications (similar to our patient) who presented with left hemiparesis and was subsequently found to have a SCEH at the level of C3 through first thoracic (T1).^6^ Approximately, 25–70% of patients diagnosed with spontaneous spinal epidural hematomas have been reported to be taking an anticoagulant medication.^7^ In contrast to the cases discussed above, Salehpour et al reported a case in 2018 of a 31-year-old male diagnosed with SCEH who was without significant past medical history or use of antithrombotic medications.^8^ Although various causes have been attributed to SCEH, including the use of antithrombotic medications, in as many as 40–50% of the cases the exact cause remains unknown.^2^,^9^ It is likely that the daily low-dose aspirin played a role in our patient.

SCEH are a dynamic process that often begin with localized neck pain as blood collects in the epidural space, and can progress to paresthesia, paraplegia, quadriplegia, or hemiparesis as the hematoma expands and subsequently compresses the spinal cord.^2^,^9^ This is the same progression we witnessed in our patient. Taha et al described a 41-year-old man diagnosed with SCEH who presented with six days of neck pain radiating to both upper extremities that subsequently progressed to quadriparesis and urinary urgency.^4^ Further complicating the clinical picture of SCEH, Hongo et al described two case reports of elderly Japanese men diagnosed with SCEH who presented with sudden onset of ataxic gait, rather than the more commonly described neck pain and associated progressive neurologic deficits.^10^

EPs are often challenged to identify patients with a SCEH who have a presentation that may mimic other, more common diagnoses, such as TIA or acute ischemic stroke.^9^,^11^,^12^ The misdiagnosis of a SCEH as a TIA or ischemic stroke could lead to the patient receiving antithrombotic medications, which could worsen the hematoma expansion and, ultimately, adversely affect the patient’s morbidity and mortality. In just such a situation, Morimoto et al described 71-year-old male who presented with sudden onset of neck pain and left hemiparesis, who received tissue plasminogen activator for suspected ischemic stroke, with subsequent worsening in neurologic function. The patient was ultimately diagnosed with SCEH on further work-up and imaging.^13^

MRI of the spine is the imaging modality of choice for identifying a SCEH.^6^,^14^ According to Matsumura et al, the hematoma appears on MRI as an isointense signal on T1-weighted images within 24 hours of symptom onset and as a hyperintense signal on T2-weighted images after 36 hours.^14^ Obtaining a stat MRI of the cervical spine shortly after patient arrival to the ED may prove to be difficult depending on resource availability; however, the information provided is invaluable for the diagnosis and guidance of surgical intervention.

Emergency surgical intervention is recommended as the primary treatment for SCEH, given concern for possible irreversible and permanent neurologic damage.^12^,^15^ A retrospective review of 35 cases of spontaneous spinal epidural hematoma by Liao et al reported that patients who presented with less severity or duration of neurologic deficits had better neurologic outcomes after surgical intervention.^15^ Our patient did extremely well, with no residual neurologic deficits following surgical intervention.

## CONCLUSION

Spontaneous cervical epidural hematoma is an uncommon yet “cannot miss” neurologic emergency that EPs should consider in the differential diagnosis for patients presenting with acute neck pain, with or without signs of cord compression.

## Figures and Tables

**Image:**
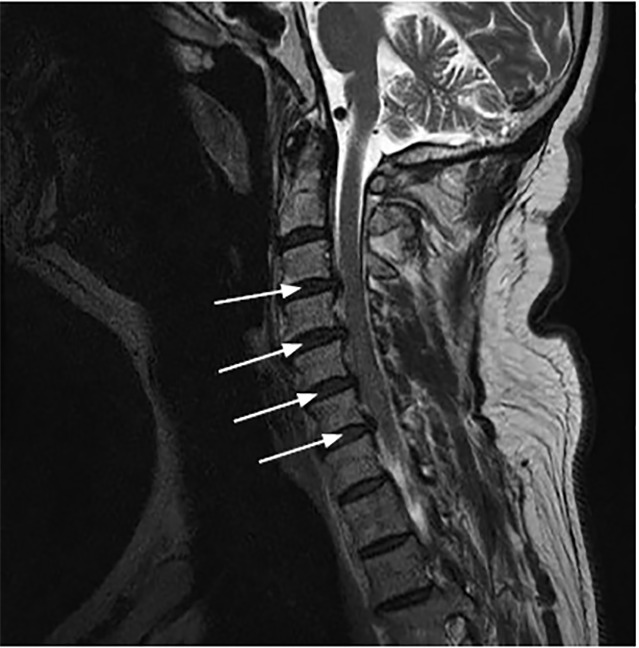
Epidural hematoma in left posterolateral spinal canal extending from mid C3 to mid C7 level (arrows). The fluid displaces the cord anteromedially and there is multilevel deformity.
